# Predicting SARS-CoV-2 Infection Trend Using Technical Analysis Indicators

**DOI:** 10.1017/dmp.2020.254

**Published:** 2020-07-17

**Authors:** Marino Paroli, Maria Isabella Sirinian

**Affiliations:** Department of Clinical, Anesthesiologic, and Cardiovascular Sciences, Sapienza University of Rome, Italy

**Keywords:** immunization, medical records, Internet

## Abstract

**Objectives:**

Coronavirus disease 2019 (COVID-19) pandemic is a global health emergency caused by severe acute respiratory syndrome coronavirus 2 (SARS-CoV-2). This study aimed to evaluate whether technical analysis (TA) indicators, commonly used in the financial market to spot security price trend reversals, might be proficiently used also to anticipate a possible increase of SARS-Cov-2 spread.

**Methods:**

Analysis was performed on datasets from Italy, Iran, and Brazil. TA indicators tested were: (1) the combined use of a faster (3-d) and a slower (20-d) simple moving averages (SMA), (2) the moving average converge/divergence (MACD), and (3) the divergence in the direction of the number of new daily cases trend and the corresponding MACD histogram.

**Results:**

We found that the use of both fast/slow SMAs and MACD provided a reliable signal of trend inversion of SARS-Cov-2 spread. Results were consistent for all the 3 countries considered. The trend reversals signaled by the indicators were always followed by a sustained trend persistence until a new signal of reversal appeared.

**Conclusions:**

TA indicators tested here proved to be reliable tools to identify in the short mid-term a subsequent change of direction of viral spread trend either downward, upward, or sideward.

Severe acute respiratory syndrome coronavirus 2 (SARS-CoV-2) is the etiological agent of coronavirus disease 2019 (COVID-19).^1^ At the time of this writing, COVID-19 pandemic is a global emergency with more than 400,000 deaths worldwide. With no effective antiviral drugs and no vaccines available, prevention of COVID-19 relies upon detection and isolation of symptomatic cases and restrictive mass quarantines.^2^ Mass quarantine, however, poses a serious risk of a second crisis in the form of an economic recession.^3^ Therefore, governments are cautiously allowing the progressive come back to work without certainty that further infection waves will not occur. Different epidemiological models are used to forecast in real-time the number of new cases and identify possible pandemic outbreaks. These include compartmental models, agent-based models, and metapopulation models.^4,5^ However, any epidemiological model has its own limitation, and the addition of new predictive tools is desired.

Technical analysis (TA) is a methodology used in the financial market to forecast the direction of prices of different instruments, such as futures, commodities, indices, and stocks. TA is based on the idea that identification of previous price changes provides an accurate prediction of future price trajectories by using historical price charts.^6^ TA may evaluate monthly, weekly, daily, and even intraday data. This methodology is, therefore, useful for a mid-short-term forecast. TA uses different mathematical indicators that generate signals to buy or to sell financial security indicating that a trend reversal is likely to occur. More frequently used indicators are the use of simple moving averages (SMAs) and the moving average convergence/divergence (MACD).^7,8^ In more detail, SMA calculates the average of a range of closing prices by several periods in that range. A common use of SMA is to combine a pair of SMAs with each covering different time frames. If the shorter-term (fast), SMA crosses a longer-term (slow) SMA from above, an uptrend is expected. On the other hand, if the slow SMA is crossed by the fast SMA from below, a downtrend might be expected.

MACD is an indicator calculated by subtracting a longer-period exponential moving average (EMA) from a shorter-period EMA. This calculation results in the MACD line. A 9-d EMA of the MACD (signal line) is then plotted on top of the MACD line. These lines oscillate above and below the zero-line, and their crossover works as a signal of a trend reversal. Additionally, the difference between the 2 lines is plotted as a histogram that oscillates above and below the zero-axis (MACD histogram). Divergences of a trend between raw data and the corresponding MACD histogram function as a sign of trend weakening or anticipate a trend reversal. Here, we examined how the combined use of a fast and a slow SMA and the use MACD can predict the direction of SARS-CoV-2 spreading, using the daily new cases of infection freely available in official sites on the Web as dataset. The analysis was performed on the data concerning Italy, Iran, and Brazil.

## METHODS

The data of daily new confirmed cases were extracted from the European Union (EU) Open Data Portal available online at https://data.europa.eu/euodp/en/home. Analyzed data ranged from April 20, 2020, to May 31, 2020. TA indicators used in this study included the following.

### Combined Use of SMAs

A fast (3-d period) and a slow (20-d period) SMAs were used. SMAs were calculated according to the following formula: SMA = (Data_1_ + Data_2_ +…Data_n_)/n were Data_n_ = number of new daily infections at period n and n = the number of data period (in our case 3 and 20 d, respectively). The crossing of the slow SMA by the fast SMA from above or below indicated a trend reversal to the downside or the upside, respectively.

### MACD

The MACD line was obtained by subtracting a 26-d EMA from a 12 d EMA. EMAs were calculated according to the following formula: EMA = Data(t) × k + EMA(y) × (1 – k) where Data are the number of new daily infections, t = today, y = yesterday, k = 2/(n+1) where n = number of days in EMA (in our case 26 and 12, respectively). Signal-line was 9-d EMA of the MACD line. MACD histogram was calculated subtracting the signal line from the MACD line. A trend reversal to the upside was signaled when the MACD line crossed the signal line from below, while a reversal trend to the downside was signaled when the MACD line crossed the signal line from above. Later, the signal of a trend reversal was shown when the MACD line crossed the zero-axis, acting as a confirmation of the first signal.

### Divergences

Divergence is defined when the trends of raw data and the TA indicator have opposite directions. In this study, divergences were detected after drawing the trend line of the graph showing the new daily infections and the corresponding MACD histogram during the same time frame. Trend lines were drawn above the data in the downtrends, and below the data in the uptrends.

All TA indicators were calculated according to the appropriate formulas using Microsoft Excel software.

## RESULTS

**FIGURE 1:**
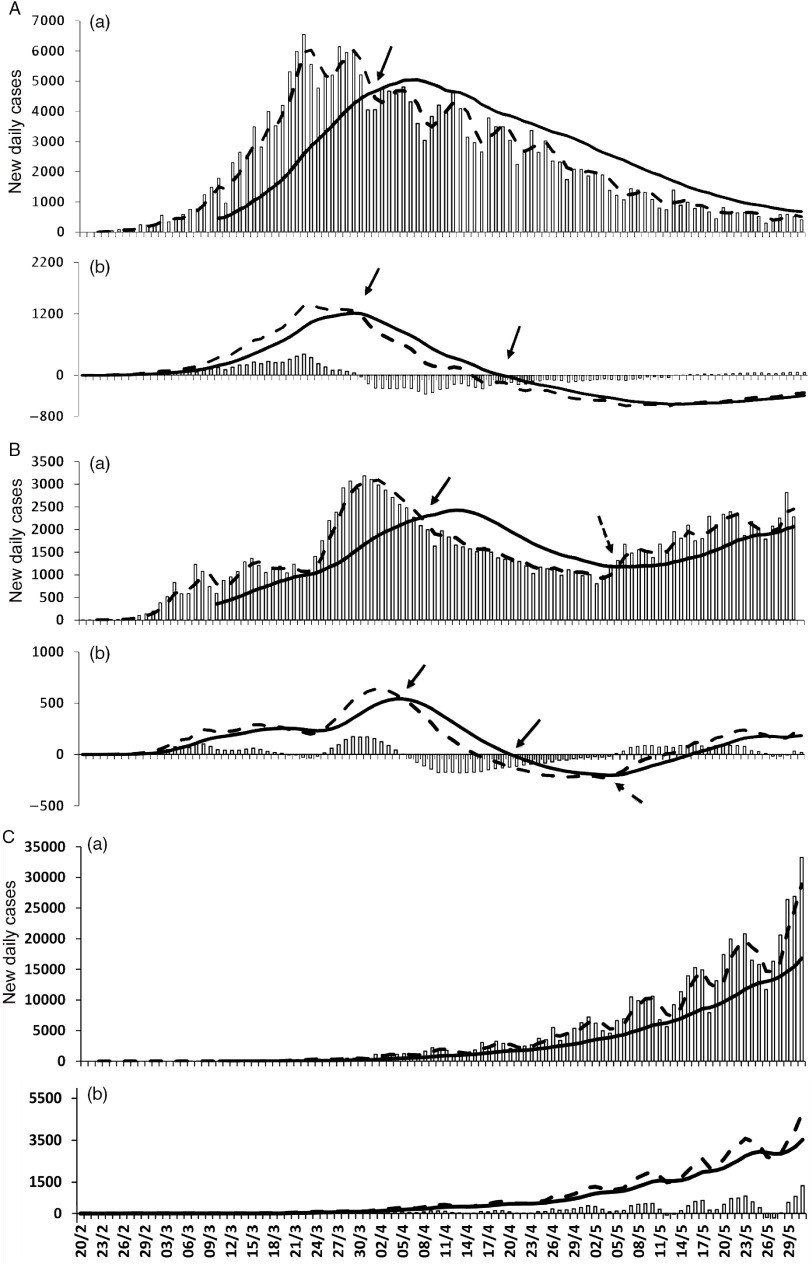
Panels A, B, and C Show Data From Italy, Iran, and Brazil, Respectively. In graph (a) of each panel, new daily infections (open bars), fast SMA (dotted line), and slow SMA (solid line) are shown. In graph (b), the MACD line (dotted line), the signal line (solid line), and MACD histogram (open bars) are shown. Trend reversal is indicated by solid arrows (downward) or dotted arrows (upward).

**FIGURE 2:**
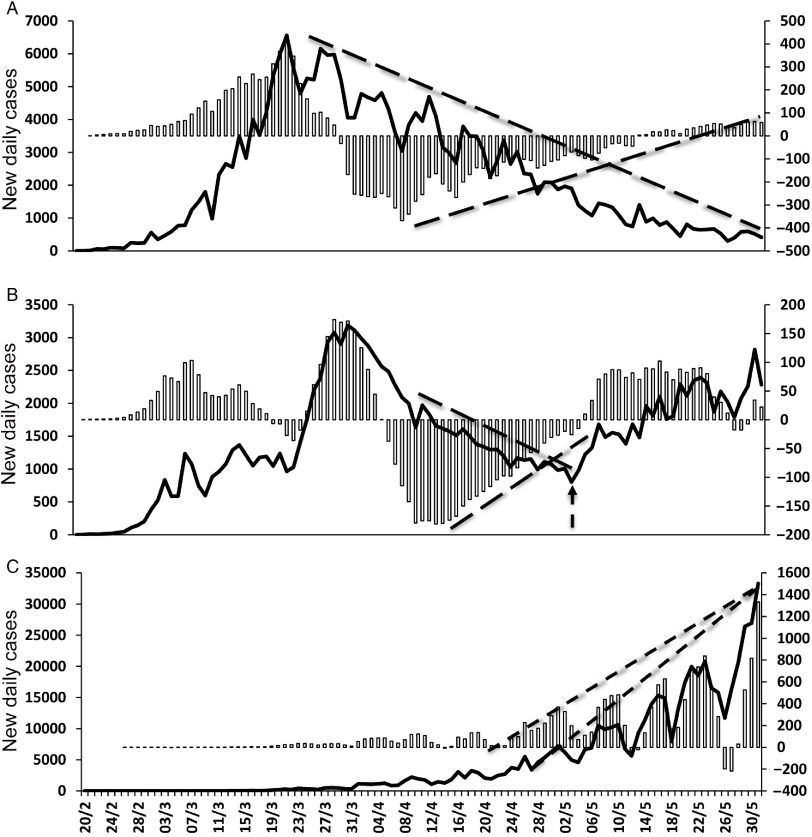
Panels A, B, and C Show Data From Italy, Iran, and Brazil, Respectively. In each panel, new daily infections are shown as a solid line, MACD histogram as open bars. Trend lines are represented as dotted lines. In panel B, the dotted arrow indicates a trend reversal to the upside.

Trend analysis of daily new infections in Italy (Figure 1A (a)) showed that the 3-d SMA crossed the 20-d-SMA line from above on April 3, 2020, indicating that the upward trend was likely to change to the downside. Thereafter, the number of new daily cases continued to decrease, indicating that this signal was highly reliable to predict a sustained trend reversal to the downside. MACD analysis (Figure 1A (b)) showed a similar signal a little early on April 1, 2020, later confirmed by the cross of zero-axis by the MACD line. A divergence between trends of daily cases and the MACD histogram was visible in Italy (Figure 2A) from April 8, 2020. The trend to the downside continued without any evident change of direction, although it seemed to be weaker than before and continuing in a sideways direction.

In Iran, as indicated in Figure 1B (a), the first signal of a trend reversal to the downside appeared on the graph on April 8, 2020. This signal was followed by a consistent reduction in the number of daily infections, which lasted almost a month. Thereafter, on May 5, 2020, a signal of a new and opposite trend reversal was shown, followed by a progressive upward trend. MACD indicator (Figure 1B (b)) also signaled the 2 opposite trends. The first downward trend was noticeable on April 5, 2020, whereas the upward reversal sign appeared on May 3, 2020. A divergence between the number of new daily cases and MACD histogram (Figure 2B) was followed by a clear upward reversal trend from May 1, 2020 on. Data from Brazil (Figure 1C (a) (b)) do not show any trend reversal. In the case of Brazil, the trend of the novel daily cases was constantly directed upward. As a confirmation, no divergence between trend lines occurred (Figure 2C).

## DISCUSSION

Technical analysis is a means to examine and forecast how financial instruments trend. TA is based on the idea that, if previous market patterns can be identified, a fairly accurate prediction of future price trajectories can be made. This is obtained by the use of mathematical tools defined as market indicators.

The TA approach is opposed to fundamental analysis (FA), which focuses on the intrinsic value of a specific asset for future investments. In FA, daily prices are ignored, the attention is entirely focused on the balance sheet, strategic initiatives, microeconomic indicators, and consumer behavior.^6^ Therefore, TA but not FA, is a methodology potentially usable outside the financial market being based on data trends.

In this regard, a combination of fast and slow SMAs and MACD are among the most commonly used technical indicators in the financial market. These indicators are particularly appreciated for their sensitivity to predict trend reversals and for the ease of their use. We, therefore, tested if both indicators would be potentially useful to detect SARS-CoV-2 epidemic trend reversals using the number of daily reported new confirmed cases as data instead of prices. We found that combined SMAs and MACD were capable to signal prompt trend reversals, which were then confirmed by subsequent and lasting trends in the predicted direction. The reliability of TA indicators was confirmed by analyzing data from either Italy, Iran, or Brazil. We took these 3 countries into consideration because the health system reacted to the pandemic in different ways. In Italy, a mass quarantine was prescribed soon after the awareness of the COVID-19 outbreak. In Iran, after an initial period of lockdown, a precocious lift of the restrictive measures was decided due to economic reasons. In Brazil, although the increase of new cases was constant within the observation period, no mass quarantine was instituted.

It is worth noting that, although TA tools proved to be highly reliable and sensitive in this study, this methodology may not always apply to epidemiology. In particular, it can help to predict the trend of the epidemic, ie, the epidemic curve, based on historical data, but it does not offer an explanation to other epidemiological questions. In the case of SARS-CoV-2 infection, TA cannot provide any information on viral biology, the efficiency of preventive behaviors or population immunological status.

A major limitation of this study is the intrinsic nature of the data. The reported number of daily new cases is partially inaccurate because a consistent number of new infections can be missed due to the presence of asymptomatic subjects.^9^ Underreporting of cases due to several factors is another major limitation of any epidemiological model. Moreover, the number of diagnostic tests taken daily may fluctuate significantly. Finally, the incidence of infection can vary among areas of the same country.^10^ Nevertheless, we suggest that TA indicators might provide reliable real-time information on how SARS-Cov-2 infection is spreading to prepare adequately and advance new containment measures if needed.
